# Effects of Human Presence and Voice on the Behaviour of Shelter Dogs and Cats: A Preliminary Study

**DOI:** 10.3390/ani11020406

**Published:** 2021-02-05

**Authors:** Adele Tuozzi, Christine Arhant, Kristina Anderle, Jessica Backes, Catherine Cords, Viola Magierski, Jean-Loup Rault, Ines Windschnurer

**Affiliations:** Department for Farm Animals and Veterinary Public Health, Institute of Animal Welfare Science, University of Veterinary Medicine, Veterinärplatz 1, 1210 Vienna, Austria; kristina.anderle@gmx.at (K.A.); jess.backes@hotmail.com (J.B.); cathy.cords@gmx.de (C.C.); violamagierski@gmail.com (V.M.); Jean-Loup.Rault@vetmeduni.ac.at (J.-L.R.); Ines.Windschnurer@vetmeduni.ac.at (I.W.)

**Keywords:** animal shelter, auditory, contact, enrichment, human–animal interaction, welfare

## Abstract

**Simple Summary:**

Environmental enrichment is fundamental for the welfare of shelter animals. This study compared the behaviour of single-housed shelter dogs and cats while being read a book (using a prerecording) in the presence of an unfamiliar human (without direct physical contact) or in its absence. Behaviours such as scratching the door, gaze direction and location in relation to the audio source/human were observed for 10 min. When a human was present, dogs spent longer in their bed and looking at the auditory source, while cats scratched the door and rubbed against the door. In the absence of the human, cats tended to spend more time in the vertical dimension, where the hiding boxes were located. Overall, the presence of a person, combined with auditory enrichment, induced greater interest compared to just an audio stimulation in both shelter dogs and cats. However, some animals showed signs of frustration likely due to not being able to physically interact with the human. Given that dogs that exhibit calmness and cats that scratch the door in the presence of humans and try to attract human attention are more likely to be adopted, auditory enrichment combined with human presence promotes behaviours that may consequentially increase adoption rate.

**Abstract:**

Reading books to shelter animals combining auditory enrichment with human presence is increasingly used although its effects on animal welfare have not yet been investigated. This study compared the behaviour of single-housed shelter dogs and cats during a prerecorded reading condition in the absence or presence of an unfamiliar human (without direct physical contact). Fourteen dogs and twenty-one cats were observed in their enclosure in the two conditions in a counterbalanced order. Behaviours such as scratching the door, gaze direction and location in relation to the audio source/human were analysed from video recording for 10 min per condition. Dogs spent more time in their bed (*p* < 0.047) and looking at the auditory source (*p* < 0.004) when a human was present. Cats showed door scratching and rubbing when a human was present (*p* < 0.043), whereas they tended to spend more time in the vertical dimension (*p* = 0.051), where the hiding boxes were located, during auditory stimulation without a human present. These results show that the presence of a human induces greater interest compared to just audio stimulation in shelter dogs and cats but may induce frustration likely due to not being able to physically interact in some animals.

## 1. Introduction

Shelters can be a highly stressful environment for dogs and cats due to the disruption of former social relationships, social isolation, confinement, exposure to unfamiliar humans and animals and unpredictable events [1,2]. This situation is typically stressful for dogs and cats, as shown by elevated levels of cortisol [3,4,5]. Environmental enrichment can be an effective way to improve the welfare of these animals by mitigating the effect of stress caused by the shelter conditions [4,6]. Enrichment in shelters can come in different forms and includes animate enrichment, such as contact with humans or conspecifics, and inanimate enrichment, such as altering the physical environment around the animals by providing objects to play with or sensory enrichment like auditory and olfactory stimulation [7,8,9,10]. In the case of dogs, toys and cage furniture seem to play an important role, particularly when those are presented as new stimuli to break the monotony of the kennel [11]. Interactions with humans such as stroking or play, but also passive human presence, can decrease the stress levels caused by a novel environment in dogs [12,13]. For cats, offering space dedicated to hiding and climbing and scratching surfaces was shown to be particularly beneficial [14], and cognitive enrichment also has positive effects on cat behaviour and welfare [10,15,16]. In general, environmental enrichment in a shelter environment should allow the animals to perform species-specific behaviours that fulfil their needs, helping them to cope with the stressors of the shelter and reduce abnormal and stereotypic behaviours performed as a consequence of stress [17].

Auditory stimulation as a means of sensory enrichment has received little attention in comparison to social and physical enrichment [8]. Auditory enrichment may come in different forms, e.g., as sounds of conspecifics, music or environmental sounds [17,18]. Human voice can also be stimulating to animals, but only few studies have investigated its effect as auditory enrichment and its influence on shelter animals. Gentle stroking combined with human vocalisation (“high pitched gentle tone”) were shown to reduce stress and promote mucosal immunity [19], to reduce the incidence of upper respiratory tract disease [19], and to decrease fear of humans in cats [5]. However, playbacks of human conversation did not change shelter dogs’ behaviour [20]. Another way to implement the human voice as sensory stimulation is the use of audiobooks, i.e., audio recordings of someone reading a book, and to play them to shelter animals. Audiobooks can influence the behaviour of shelter dogs, inducing a stronger calming effect compared to other forms of auditory enrichment such as classical music [21]. The effects of audiobooks on the behaviour of shelter cats have not yet been explored. Recently, reading to shelter dogs and cats (combining auditory stimulation and human presence) has been increasingly popular in animal shelters (e.g., [22,23]). Although the effects of reading to dogs on the reading skills of children have been investigated [24], its effects on the animals’ behaviour have not been addressed so far. The presence of unfamiliar people in shelter dog kennels causes high levels of barking, decreased resting behaviour and increased levels of stereotypes [25]. Therefore, there is a need to compare the effects of auditory stimulation with a human present to auditory stimulation without a human present, which has been shown to induce positive behaviour changes in dogs [21]. Hence, the aim of this pilot study was to explore the effect of human presence during the playback of an audiobook on the behaviour of shelter dogs and cats. 

## 2. Animals, Materials and Methods

Ethical approval was obtained from the Animal Ethics Committee of the University of Veterinary Medicine, Vienna (protocol number 05/09/2018) in accordance with guidelines for Good Scientific Practice and with national legislation. 

### 2.1. Animals and Housing

Both dogs and cats were housed in the same Austrian shelter. This shelter can house up to 150 dogs and 300 cats. As a no-kill policy is legally required in Austria, animals difficult to rehome are long-term housed [26]. Animals participating in the experiments were selected based on availability in the adoption section of the shelter. All animals were in good health and no deaf animals were included. The dogs and cats’ enclosures were located in the same building but in separate areas. Fourteen dogs (11 males and 3 females) aged between 11 months and 10 years (mean age: 3.7 years; median: 4.5 years) were included in the experiment. The dogs had been kept in the shelter for 1 month to 2 years (mean length of stay: 5.5 months, median: 2 month). No dog had a docked or corkscrew tail. The dog kennel section always contained two rows of kennels facing each other, divided by a working corridor. The participating dogs were kept in individual kennels, consisting of an indoor (either 5 m^2^ or 5.8 m^2^) and an outdoor section (10 m^2^). Each indoor section had a tiled floor and a glass front including partly frosted glass to have some visual protection (square section mostly at the dog bed level). Every kennel contained a dog bed with soft bedding, in most cases a chew toy, and some kennels were equipped with a second lying place or another furniture. Dogs were provided with water *ad libitum*, fed twice daily and allowed to leave their kennel once daily either for a walk or stay in a large outdoor run, always outside of testing sessions. Outdoor sections were not accessible at the time of testing.

In the cat experiment, 21 cats (14 males and 7 females) aged between less than 1 year and 14 years (mean age: 3.5 years; median 1.0 year) were included. The information on the exact duration of their stay in the shelter was not available, but all cats were in the shelter for a minimum of three weeks. All cats were housed individually. Each cat enclosure measured 2 m in length, 1.50 m in width and 1.96 m in height, and was equipped with a food and a water bowl, a chair, a hammock, toys, a blanket, a hiding box, shelves/platforms attached in different heights to the wall and a litterbox. The cat enclosures were located in the same section in three different aisles: one aisle with two rows of enclosures facing each other, divided by a working corridor, and the cats could see each other through a Plexiglas door; and in the other two aisles, the enclosures did not face each other. Cats were provided with water *ad libitum* and fed twice daily.

### 2.2. Experimental Design

The dog experiment was carried out by two female experimenters and the cat experiment was carried out by three other female experimenters. Each experimenter tested approximately the same number of animals. Each animal was tested in two different conditions during which an audio recording of an unfamiliar female voice reading a book (audiobook) was played to the animals, once in the presence of an unfamiliar person (P+) and once in the absence of a person (P−). The two conditions were carried out in a counterbalanced order. The first assignment was made to condition P− and the order of the conditions was switched after every animal. It was not feasible to include additional baseline or control conditions because of a limited time frame available for testing at the shelter. Each dog and cat was tested in his/her own enclosure. Experimenters did not enter the enclosure or have physical contact with the animal. A portable speaker set up in front of the enclosure was used to play the audiobook recording in both conditions P+ and P−. The recorded text was from the book “Manchmal wär ich gern mein Hund” (translated as “Sometimes I would like to be my dog”) by Rita Pohle (2014) [27]. It was prerecorded with a microphone (Boya Lavalier-Microphone BY-M1) connected to a smartphone (Samsung X-Cover 2) and a voice recorder (summer.mobile.apps) by the experimenters (thus, always female voices; only German native speakers: two in the dog, and one in the cat project). In the condition with the person present (P+), the respective experimenter mimicked the act of reading (sitting down, gazing at the book, moving the lips, and turning pages) and only the “Reader” experimenter was visible to the animals. Due to different housing situations (different design of the enclosures) and different species-specific behaviours, the two experiments were conducted with some differences in methodology. Before the start of a testing session, both dogs and cats received a habituation phase to get used to the presence of the speakers and the person in case of a P+ condition. This habituation to the respective experimenter lasted 3 min for the dogs and 2 min for the cats. In addition, for the dogs, both experimenters sat down in different areas of a kennel section for a total of ten minutes to accustom the dogs to their presence before the start of the testing sessions on that day. This additional time for habituation was chosen because of the high level of arousal in dogs in reaction to the arrival of an unfamiliar human in the kennel building. After the habituation phase, the actual testing session started (either P+ or P−) and lasted for 10 min. 

On each testing day, five to six dogs were tested between 13:00 h and 17:00 h undergoing both conditions on the same day. Between the two conditions, the dogs remained in their kennel but could usually not see the human. The second condition followed half an hour to one hour after the first condition. In the P+ condition, the person “reading” to the dog sat behind the speaker directly in front of the kennel.

The cats were tested between 1000 h and 1500 h, with half an hour to one hour between the two testing conditions. The person “reading” during the condition P+ was sitting sideways next to the glass door, which constituted the front of the enclosure, but not in front of it in order to avoid blocking the camera’s field of view. However, the person was still visible to the cats.

We never tested animals from adjacent enclosures or in close proximity during the same testing sequence, but we cannot rule out that some animals could hear parts of the recording or could see the human from a distance during the testing session of another individual.

All sessions were filmed with a video camera ( ) placed on a tripod at a distance of 1 m from the enclosure.

### 2.3. Recorded Behaviours

**Figure 1:**
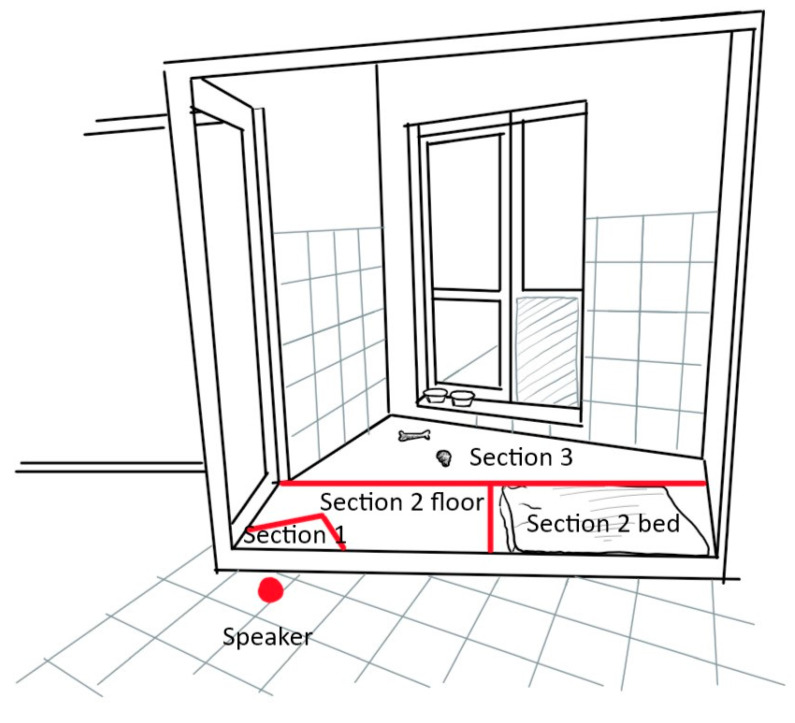
Representation of the dogs’ kennel enclosure with the different sections. In the P+ condition the reader was always sitting directly behind the speakers facing towards the kennel.

For dogs, the behaviour and location in relation to the auditory source was recorded using an ethogram (Table 1). While most behaviours were recorded as durations in seconds, short lasting behaviours were recorded as frequencies (barking, lip licking, yawning, shaking and stretching). Since kennels differed slightly in size, the indoor kennel was split in four sections to evaluate the distance from the auditory source (Figure 1).

**Figure 2:**
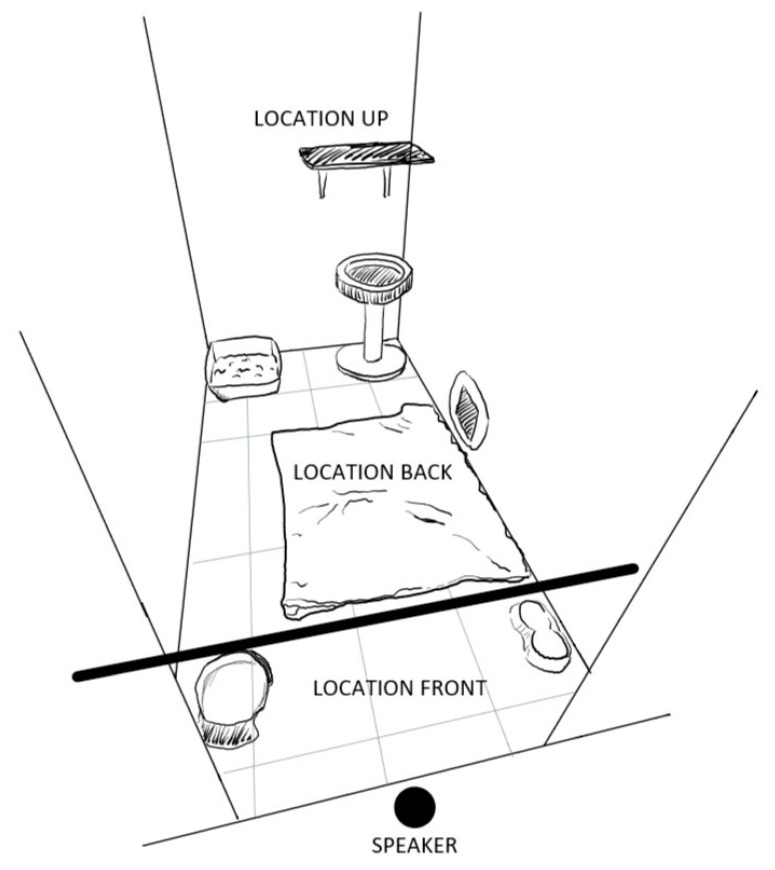
Representation of the cats’ enclosure with the different sections (chair and hammock not depicted in order not to obstruct the view). In the P+ condition, the reader was always sitting left from the speaker facing sideways visible to the cats but not obscuring the front door and the camera’s field of view.

For cats, the behaviour and location in relation to the auditory source was recorded using an ethogram (Table 2, Figure 2). While most behaviours were recorded as durations in seconds, short lasting behaviours were recorded as frequencies (hissing, meowing and yawning).

#### Coding and Inter-Rater Reliability Assessment

For both experiments, the videos were coded with the software Solomon Coder (Version: Beta 17.03.22). Overall, three persons (two of them were also performing the experiments) coded dog behaviour and three persons (all of them also performed the experiments) coded cat behaviour. 

During the training of the coders, the inter-rater reliability assessment was carried out. For dogs, 3 min sequences from 15 videos were used and for cats, 5 min sequences from 12 videos were used. To assess agreement between coders, Spearman correlation coefficients and Wilcoxon tests were performed on every possible pairwise combination.

For the final analyses in both species, one coder always coded both conditions for the same animal.

### 2.4. Data Analysis

The statistical analyses were conducted using SPSS (SPSS Statistics for Windows, version 25.0, IBM Corp., Armonk, NY, USA). Data were analysed separately for dogs and cats. Some behaviours were not or very rarely recorded (Table 3), or did not reach satisfactory inter-rater reliability (criteria were a correlation of r_s_ ≤ 0.7 and a non-significant Wilcoxon-test but see Section 3.1), and therefore these behaviours were omitted from further analyses.

Whereas the dogs’ behaviour remained visible almost all the time, the cats’ behaviour was sometimes not visible, e.g., when in the box (dogs: person present in seconds: all dogs fully visible except for one dog out of sight for 6.2 s; person absent in seconds: all dogs fully visible except for the same dog out of sight for 1.4 s; cats: person present (P+) in minutes: mean: 8.87, median: 9.91, min: 2.91, max: 10.00; person absent (P−) in minutes: mean: 8.82, median: 9.99, min: 4.6; max: 10.00). Therefore, data were calculated as the percentage relative to visible observation time and, for event behaviours, frequencies per minute based on visible observation time. In addition, oral behaviours of dogs were corrected for the time the dog’s face was not visible, and solely barking, which was always visible, were analysed as raw frequency per minute, whereas events of yawning and lip licks were first adjusted for the time the dogs face was visible before calculating the frequency per minute.

According to Shapiro Wilks tests, data were not normally distributed. Thus, Wilcoxon tests were performed to compare the behaviours of the individual cats and dogs between the P+ and the P− conditions.

To further explore dog and cat behaviour during P+, we calculated Spearman correlations of behaviours differing significantly between the two conditions and all other behaviours within the P+ condition. Statistical differences with *p* ≤ 0.05 are referred to as statistically significant, and *p* ≤ 0.1 are interpreted as a tendency. Due to the exploratory nature of the study and the relatively small sample size, we refrained from correcting for multiple testing. In exploratory studies, the risk of type II errors, leading to failure to report potentially significant results, is considered more relevant than that of type I errors [32]. When data are depicted as box plots, the bold line in the boxes corresponds to the median, the lower and upper line of the grey box to the first and third quartile; the whiskers represent the lowest and highest values that are still within a range of 1.5 × interquartile range. Outliers (all values between 1.5 × interquartile range and 3 × interquartile range) are marked with a circle, and extreme values (outside of a range of 3 × interquartile range) with an asterisk.

## 3. Results

### 3.1. Inter-Rater Reliability

Inter-rater reliability between the three observers was very good to acceptable for the dog behaviour analyses (Spearman correlation coefficients: mean r_s_ = 0.94, min r_s_ = 0.621, max r_s_ = 1.000). To control also for systematic errors between the observers, Wilcoxon tests were performed. Five Wilcoxon tests had a *p*-value smaller than 0.05 (mean *p* = 0.455; min *p* = 0.012; max *p* = 1.000). However, correlations were r_s_ > 0.8 for four of these behaviours (Section 1, Section 2 floor, Section 3 and ears back), only one had also a borderline correlation coefficient of r_s_ = 0.621 (exploring). Finally, approaching had to be excluded because of insufficient agreement (r_s_ = 0.31). For the cat study, the interobserver reliability between the three observers was considered satisfactory (Spearman correlation coefficients: mean r_s_ = 0.991, min r_s_ = 0.915, max r_s_ = 1.000). Regarding Wilcoxon tests, all *p*-value were above 0.05.

### 3.2. Dogs’ Behavioural Response

In general, the dogs spent most of the time, on average two thirds, in Section 2 (more than 1 m away from the person/auditory source), and the most common behaviour was lying attentively (approximately half of the observed time). Descriptive statistics of the observed behaviours are reported in Table 4.

**Figure 3:**
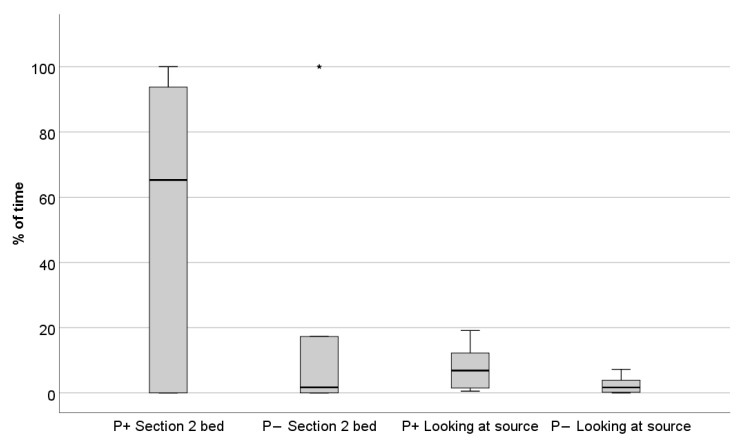
Percentage of time spent by the dogs in the Section 2 bed and the percentage of time spent by the dogs looking at the audio source in the two conditions (P+: auditory enrichment with a human present, sitting behind the speakers; P−: auditory enrichment without human presence). Section 2 bed was located in the front part of the enclosure. The dogs spent significantly more time in the Section bed 2 in the presence of the human (Wilcoxon test: *p* = 0.047) and spent significantly more time looking at the audio source when the human was present and mimicking reading (Wilcoxon test: *p* = 0.004). * depicts outlier.

Dogs spent a higher amount of time in their bed when the recording was played with a person present than when no person was present (Z = −1.988, *p* = 0.047, Table 4, Figure 3). Dogs also spent more time looking at the auditory source when a person was present compared to when no person was present (Z = −2.919, *p* = 0.004, Table 4, Figure 3). No other significant differences were found when comparing dog behaviours between the two testing conditions (all other *p*-values > 0.05, see Table 4). The mean rate of barking was once per minute in both testing conditions.

To explore whether there were behaviours that co-occur with those that differed between conditions we calculated Spearman correlations. Correlation analyses within the reading condition in the presence of the human (P+) showed that dogs that looked at the auditory source for a longer amount of time panted (r_s_ = 0.72, *p* = 0.004, Table 5) and licked their lips significantly more often (r_s_ = 0.58, *p* = 0.030; Table 5). Overall, three dogs panted and eight dogs licked their lips in the presence of the human (P+; Table 3). Dogs that remained for longer in Section 2 bed also spent significantly less time in Section 2 floor (r_s_ = 0.62, *p* = 0.019) and less time standing (r_s_ = 0.58, *p* = 0.035, Table 5).

### 3.3. Cats’ Behavioural Response

**Figure 4:**
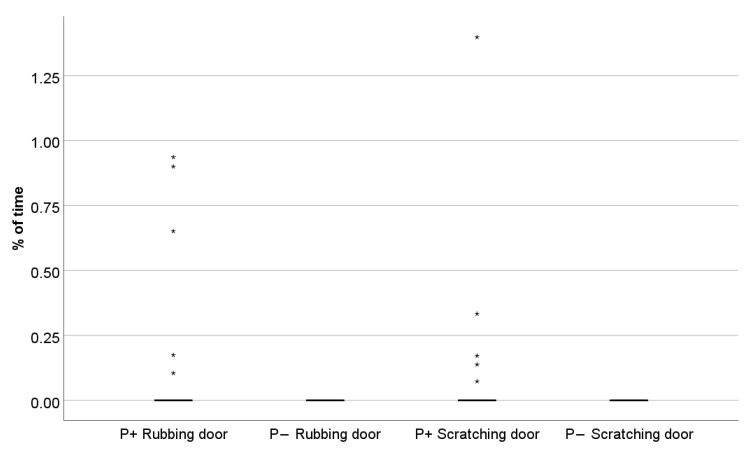
Percentage of time cats scratched and rubbed the door in the two conditions (P+: auditory enrichment with a human present; P−: auditory stimulation without human presence). The cats showed door scratching (Wilcoxon test: *p* = 0.043) and rubbing (Wilcoxon test: *p* = 0.043) only in the presence of the human (Wilcoxon test: *p* = 0.043). * depicts outlier.

**Figure 5:**
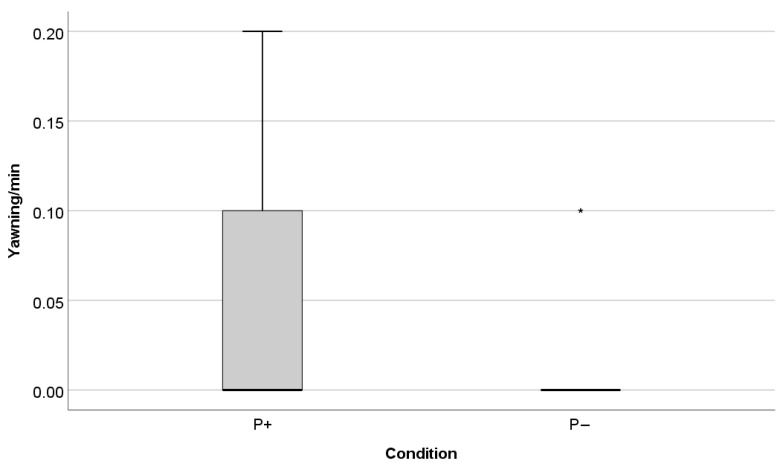
Frequency of yawning per minute in the two conditions (P+: auditory enrichment with a human present; P−: auditory enrichment without human presence). The cats showed the behaviour significantly more often in the presence of the person (Wilcoxon test: *p* = 0.018). * depicts an outlier.

In general, the cats spent most of the time in the front location, on average half of the time, and the most common basic activity was sitting (on average 40% of the observed time). Descriptive statistics of the observed behaviours are reported in Table 3 and Table 6. Cats yawned more often and only rubbed and scratched the door when the recording was played while a person was present compared to when no person was present (rubbing door: Z = −2.023, *p* = 0.043, Figure 4; scratching door: Z = −2.023, *p* = 0.043, Figure 4; yawning: Z = −1.371, *p* = 0.018, Figure 5; Table 6). Cats also tended to meow more often in the presence of the person than when no person was present (meow: Z = −1.779, *p* = 0.075), and tended to spend less time in the elevated location (where the hiding boxes were located) when a person was present than when no person was present (“% Location up”: Z = −1.955, *p* = 0.051). 

Correlation analysis between behaviours within the reading condition in the presence of the human (P+) showed that cats that yawed more often also spent more time in the front of the enclosure (location front r_s_ = 0.61, *p* = 0.004; Table 7) and less time in the back of the enclosure (location back r_s_ = −0.48, *p* = 0.029;). Cats yawning more often also spent more time interacting with the audio source (r_s_ = 0.62, *p* = 0.003). Cats spending more time scratching the door tried to interact and play with the person more often (r_s_ = 0.57, *p* = 0.007), and rubbing the door was positively correlated to the frequency of meowing (r_s_ = 0.57, *p* = 0.007).

## 4. Discussion

Overall, the findings suggest that the presence of a human had a greater effect on the behaviour of the dogs and cats than the playback of a recorded reading of a book per se. A study including a comparison to the baseline time-budget of the animal would provide further insights into the potential benefits of this type of stimulation.

### 4.1. Dogs’ Findings

Compared to the audiobook only condition, the dogs spent significantly more time in their bed when a human was also present, and more time looking at the audio source and human (as the human was sitting just behind the audio source). As shown in previous studies, playing an audiobook to shelter dogs can result in positive effects as dogs spent more time resting or sleeping and less time barking compared to other types of auditory enrichments [21]. In our study, after the habituation period we also found more time resting particularly when a human was present, but no differences in barking frequency. However, this preliminary study lacks a baseline condition with no sound and no human to compare the dogs’ behaviour to an unstimulated situation.

The practice of reading to animals kept in shelters has become a common form of enrichment (e.g., [22,33,34]). In the setting of this study, the presence of the person mimicking the act of reading the book appeared to have the greatest effect on the behaviour of the dogs, more so than the mere auditory stimulation of the sound playback. The dogs looked at the auditory source for longer when the “Reader” was sitting behind it. Hence, the physical presence of the person attracted the attention of the dog more so than just the audiobook recording alone. Former studies have shown that interactions with humans do benefit shelter dogs, e.g., in the form of decreased stress hormone levels [3,35]. Furthermore, confinement in kennels/shelters might lead to an increased need for social contact with humans, as it was found that shelter dogs approach unfamiliar people more often and form attachment bonds quickly [36,37]. Watching the auditory source/person was correlated positively to panting and lip licking; also, a tendency for more frequent yawning was present. Lip licking is a signal commonly used by dogs in communication towards humans, with a context-dependent meaning. It could represent an act that is part of a more complex active submissive display, or more specifically it may represent appeasement signals towards humans [38]. Lip licking has also been observed when expectation of a treat was not met and was interpreted as a sign of frustration in this context [39]. Yawning has been found to be related to lip licking during human presence and was interpreted as a social stress response [40]. Panting has been found to correlate to increased cortisol levels and may represent a sign that the dog is experiencing a stressful situation [35,41]. This suggests that the presence of the person without the possibility for direct physical interaction could lead to frustration and stress, particularly for dogs experiencing a lack of human contact due to confinement. In our study, around one quarter of the dogs displayed these behaviours.

Another significant finding was that the dogs spent an increased amount of time in their bed when the human was present. Although one interpretation could be that the lack of possibility for direct interaction resulted in frustration (see above), the increased amount of resting could on the other hand be interpreted as the presence of the “reading” person providing a calming element compared to the voice only. Furthermore, individuals reacted differently to the presence of the human. Correlations of staying in the bed with other behaviours indicate that these dogs walked and stood less. However, we found no other correlation giving hints at the emotional valence of this situation. An alternative interpretation is that the dogs had no possibility to hide or withdraw from the situation, as the flap to the outdoor section of the kennel was closed. In this experiment, all dogs except one were provided with plastic dog beds having rather high side walls. Like a litter box, this could provide a retreat for dogs experiencing fear in the presence of the human, as was shown for shelter cats that attempt to hide in or behind their litter box if no other hiding enrichment is provided [4]. However, the dogs did not show a backwards ear position for longer during the presence of the human. Therefore, this interpretation seems unlikely.

Overall, it is difficult to give a clear interpretation of the emotional valence in this mimicked “reading to dogs” experiment. Behaviours that would have been indicative of a negative emotional state such as ears back or panting did not differ between conditions. Behaviours such as cowering, a low tail position, stereotypes or avoidance behaviours either did not occur or were very rare. Barking can have very different qualities, functions or motivations, ranging from aggression to play [42] and it can be a massive problem in dog housing leading even to hearing loss [43]. The frequency of barking in our study did not differ between conditions and we recorded on average one bark per minute, which can be considered a rather low frequency in response to unfamiliar people being present in the kennel building. In the present study, human presence did not induce higher levels of barking as compared to audiobooks alone that were found to reduce barking in comparison to other types of music and no auditory enrichment [21]. In our study setup, a female human was sitting in front of the kennel and gazing at a book. Features of the human such as gender [44], its body position or gaze direction or whether the person is moving or being immobile [45] are likely to lead to different reactions in dogs. Overall, behaviours suggesting intense negative emotional states were not observed in our study. However, individual dogs might have experienced frustration from failed attempts to physically interact with the human present whereas others might have perceived the situation as calming or positively arousing.

With regard to adoptability, dogs that approach humans and remain in the front part of their enclosure are preferred by potential adopters [11,30,46], whereas dogs moving or facing away from humans stay in the shelter for longer. Overall, the effect of human presence may have been different between individual dogs, as dogs that looked at the person for longer may have experienced frustration caused by unsuccessful attempts to physically interact while other dogs may have experienced increased relaxation due to the presence of the human.

### 4.2. Cats’ Findings

Cats yawned more often and spent more time rubbing and scratching the door when the human was present. The higher frequency of yawning may indicate relaxation and be interpreted as a comfort behaviour [47], indicating a positive affective state in the presence of the human compared to the auditory condition alone. On the other hand, yawning may also be a sign of arousal, or possibly frustration due to the fact that the cats could not physically interact with the human [48]. In fact, cats that yawned more often spent more time in the front of the enclosure and spent more time interacting with the audio source that was distinctly located from the human in the cat study part [48,49]. 

Scratching and rubbing the door were performed only in the presence of the human, suggesting that these behaviours were directed towards the human. Usually, scratching is interpreted as visual and chemical communication, as in territorial marking; further it is used to condition claws but also as an attention-seeking behaviour [50]. The scratching may also have been an attempt to open the door in order to interact with the person. This is supported by the significant correlations between scratching and attempts to interact or play with the person. Given that scratching was performed only in the presence of the person, this suggests that it was a human-directed behaviour in the context of our study, and not a stress-related behaviour as observed previously in shelter cats [10], where cats scratched and pushed the paws against the enclosure door persistently in the absence of a human. This was interpreted as a sign of elevated stress, poor welfare and an attempt to escape [10]. Interestingly, it has been shown that scratching on the door in the presence of humans and trying to attract their attention can increase adoption rate of shelter cats, as cats that perform these behaviours are more prone to being adopted [51].

Rubbing behaviour could have a function of social bonding, thereby increasing the feeling of comfort and security for cats [52]. Furthermore, rubbing might have a function of marking and exploratory behaviour, may be elicited by the presence of an unfamiliar person [47]. It could also be a sign of affiliation with the person, but in this study there was no possibility for physical contact between the cat and the human. Thus, the rubbing behaviour could have been a failed attempt to affiliate. This is supported by the correlation of rubbing with meowing, which is the most common vocalisation in cat–human communication [53]. Cats also tended to meow more often in the presence of the person, possibly reflecting again a way to attract human attention given that it is a common form of attention-seeking [54,55].

In both conditions, the cats also attempted to interact with the audio speakers, which could be a sign of interest in a novel object. The sound may have acted as an additional attractive feature [56].

In the absence of the human, the cats tended to spend more time in the elevated location, in the hiding boxes and hammock, probably resting and sleeping in these locations. The percentage of time spent lying did not differ between testing conditions, but the 10 min observation time could have been too short to detect differences. Nevertheless, since cats tended to spend less time in the hiding box and hammock in the presence of the human, this suggests that the presence of the person prompted more active and affiliative behaviours than the audio condition alone. 

### 4.3. General Discussion

These findings show that the presence of a human had greater effects compared to an audio stimulation per se in both shelter dogs and cats living in an already physically enriched enclosure. For both species, the auditory source represents an attractive element, with dogs gazing at it and cats attempting to reach it. Therefore, it has potential to alleviate boredom in confined dogs and cats [57]. However, the results of the present study are not clear-cut regarding the valence of the emotional state triggered by this type of stimulation. Two-dimensional models of animal affective states include valence and arousal as core components [58]. Affective states always include a subjective component that cannot be measured directly. Measurable components are behavioural and physiological measures. Our pilot study relied on behavioural measures. However, interpretation is not necessarily straightforward and in the field of animal welfare science a lot of effort is invested to enable differentiation of affective states based on behaviour and physiology (e.g., [39,59,60,61]). In our study, behavioural indicators for intense negative emotional states were not observed, however some of the animals might have experienced frustration very likely due to unsuccessful attempts to physically interact. Even physiological measures are often difficult to interpret, e.g., heart rate or cortisol mainly reflect the arousal dimension as they increase in positively and negatively valenced contexts [62,63]. Suggested to reflect the valence dimension of emotional states is heart rate variability [62]; in particular parameters that measure the activation of the parasympathetic branch of the autonomic nervous system [59]. Therefore, further research is needed on the effects of reading to dogs and cats on their behaviour and particularly its potential to enhance their welfare. Future studies should also investigate physiological changes to gain more insight into the valence of the emotional state, e.g., by combining behavioural observations with measurements of heart rate variability. Furthermore, it would be interesting to investigate the effect of the familiarity of the human or the effect of long-term reading programs on long-term affective states.

In both dogs and cats, the presence of a human during auditory stimulation appears to increase engagement by the animal, spending more time at the front of their enclosure, paying attention for dogs and rubbing and meowing for cats. Therefore, having a human reading to the animal may yield positive effects by increasing the likelihood of adoption by visitors, but whether positive effects also occur from auditory stimulation alone remains to be investigated. Dogs that exhibit calmness are more likely to be chosen for adoption [64], whereas increased activity in the kennel could be perceived as linked to increased activity in the future home environment and for this reason considered as an undesirable behaviour by potential dog adopters. Furthermore, paying attention to humans may increase the adoptability in shelter dogs. In fact, gazing is considered important to dog–human bonds [65], whereas behaviours such as looking away are perceived as unfriendly or uninterested by potential adopters [30]. The adoptability of cats could especially be influenced by the approach behaviour of the cats towards a human [66]. Potential adopters prefer cats that are less fearful and more willing to approach the front of the enclosure [66,67], and are more friendly, thus those that are able to cope more successfully with the stressful shelter situation [7].

### 4.4. Limitations of the Study

This pilot study had several limitations. It is lacking a baseline condition and a human only condition and sample size was rather low due to reasons of feasibility (limited time frame for data collection). As this was the first study on reading to shelter animals, no sample size calculation was done before starting the study because there was no preliminary data available on which we could have based it. Therefore, only large effect sizes might have been detectable with appropriate power. The results of this exploratory study were not corrected for multiple testing and need to be confirmed by follow-up studies. The extensive ethograms included a wide range of behaviours, some of which never occurred. Non-occurrence could be due to the low sample size or the relatively short time window chosen for observations (twice 10 min). Another important factor to consider for this study is that we tested the enrichment conditions not under controlled laboratory conditions but in a shelter. This has advantages and disadvantages. A clear disadvantage was that we were not able to standardize every detail. For instance, we cannot rule out that during the testing session of another individual some animals could hear parts of the recording or could see the human from a distance. To avoid this, we never tested animals from adjacent enclosures or in close proximity during the same testing sequence. In addition, the animals were used to humans passing the corridor or spending some time in the corridor during cleaning routines and used to hearing background noise. The distinct response of the animals to the different treatments still suggests that the auditory stimulation directly in front of the enclosure or a human sitting directly in front of the enclosure had nevertheless different properties from these other types of stimulations such as seeing a human from a distance. A clear advantage of conducting the study in a shelter is that it allows one to explore the research question in a practical setting, where the actual reading activities take place. Despite these potential limitations, we believe that the results of this study provide valuable insights that can guide future studies and support shelters interested in implementing reading programs.

## 5. Conclusions and Implications

This is the first study investigating effects of reading to shelter animals. Based on the results of this pilot study, we concluded that human presence had greater effects compared to an audio stimulation per se without a human present in both shelter dogs and cats. This was mainly reflected by behaviour directed towards the human. However, the results of this preliminary study concerning the valence of the emotional state induced by this type of stimulation suggest that some animals might have experienced frustration because of failed attempts to physically interact. The results of this study are preliminary and are intended to inform future studies investigating “reading to shelter animals”. Future studies should include baseline conditions with no stimulation and a condition with human presence only. Factors such as familiarity of the person and characteristics of the animals should be included and elements to reduce possible negative effects such as the provision of a possibility to hide or withdraw or the provision of objects to chew on or engage with. Future studies should include physiological parameters such as heart rate variability. Effects on long-term affective states, i.e., mood, should also be assessed.

Based on what we found, to date our recommendations for setting up a reading program for dogs and cats are the following. It seems important that the animals are monitored, and individual differences and preferences are considered. Fearful animals should have the possibility to withdraw and hide, e.g., in hiding boxes or go in a comfortable outdoor section; animals that want to engage in physical contact should be given the possibility to interact with the humans present to avoid growing frustration due to physical barriers. Physical contact with a person is not always possible in some cases for security reasons, in others the kennel design does not allow touching the animal (e.g., when glass is used as a barrier). In dogs, providing chews/chew toys, such as ones filled with food during reading, may help to reduce frustration due to the inability to physically interact. Another solution could be to allow contact with other animals, like reading to a group kept in the same enclosure. In case of intense fear or aggression towards the reader other types of enrichment could be provided. However, reading to an animal can be part of a structured behaviour modification program. Further research on how to implement a reading program in the shelter environment in an optimized way is necessary, given the apparent added value of human presence already shown by a range of studies [1,51] for dogs and cats kept in shelters.

## Figures and Tables

**Table 1:** Ethogram to record the dogs’ behaviour: location, basic activity, stereotypies and other behaviours. Behaviours were adapted from [28,29,30].

Location and Behaviours	Definition
Location (Figure 1)	
Section 1	Dog is positioned within up to 1 m of the sound source
Section 2 floor	Dog is positioned in between the door and the opposite wall excluding its bed
Section 2 bed	Dog is positioned in the bed
Section 3	Dog is positioned between the back wall and Section 2
Out of sight	Dog is not visible (behind frosted glass), or only partly visible but not sufficiently to allow coding behaviour
**Basic activity**	
Lying resting	Head down, eyes half closed/closed, sleeping
Lying attentive	Watching the surroundings, head up or down
Walking	Dog engages in ambulatory gait around the kennel
Standing	Dogs stands on four legs immobile
Sitting	The dog’s rear is on the ground, with the rear legs tucked and the front legs extended
**Stereotypies**	
Pacing	Repeatedly walking (>3 times) around the kennel in a fixed route
Jumping on window	Repeated up and down movements (>3 times) in front of the kennel, often both front paws making contact with the front window
Circling	Moving repeatedly (>3 times) in a circle
Others	Any other repetitive (>3 occurrences) behaviour with a specific pattern
**Other behaviours**	
Approaching	Seeking contact with the sound source, e.g., sniffing, pawing, leaning against front window, jumping on window
Avoiding	Aggressive behaviour towards the sound source (e.g., growling, lunging)
Exploring	Dog is walking or standing and sniffing the environment
Looking at auditory source	Dog gazes at the speakers (and the narrator in the P+ condition)
Cowering/crouching and/or hiding	Lowered body posture, taking cover behind furniture
Self-grooming	Licking or nibbling its own skin or fur
Panting	Tongue exposed with audible and/or observable breathing
Tail lowered	Tail held low or tightly between hind legs, may be curled under genital area or ventral side
Ears back	One or both ears folded against side and/or back of the head and having a flattened appearance
Manipulating environment	Oral contact (e.g., licking, chewing, biting) toward an object in the environment, dog is stationary
Drinking	Take water into the mouth and swallow
Show back	Dog sits down with the back turned towards the auditory source
Face not visible	Face of the dog is not observable
Barking *	Vocalisation of very short duration and low frequency
Lip licking *	Moving tongue over lips/nose
Yawning *	Opens mouth widely and inhales
Shaking *	Rotation of the body starting at the head and moving caudally
Stretching *	Extend either forelegs or hind legs and hold for 1–2 s

* Recorded as events due to their short duration.

**Table 2:** Ethogram to record the cats’ behaviour: locations, basic activity and other behaviours. Behaviours were adapted from [31].

Location and Behaviours	Definition
Location	
Location front	The cat is located in the front of the enclosure, less than 50 cm from the door
Location back	The cat is on the floor of the enclosure, at a distance >50 cm from the door
Location up	The cat is on an elevated part of the enclosure (e.g., on a chair or shelf/platform, which were located in the back of the enclosure)
Location not visible	The cat is not visible from the outside of the enclosure (e.g., in the hiding box)
**Basic activity**	
Standing	The cat is in a stationary position, the four legs touching the ground and stretched
Sitting	The cat is in a stationary position with the hind legs bended and the front legs extended, or both pair of legs are bended in a crouched position (but the lateral side of the body is not touching the ground)
Lying head up	The cat’s ventral region or side is touching the ground, with the head in an upright position
Lying head down	The cat’s body is lying completely horizontally on the ground, with the head in contact with the ground
Active/in locomotion	The cat is moving, e.g., running, walking or jumping
Using the litterbox	The cat is inside the litterbox
Activity not visible	The activity of the cat is not visible from the outside of the enclosure
**Other behaviours**	
Interact with the Reader	The cat’s attempt to reach the Reader through the space between the door and the floor (excluding elements of play behaviour)
Interact with the speaker	The cat’s attempt to reach the speaker through the space between the door and the floor (excluding elements of play behaviour)
Rubbing the door	The cat moves the head/body against the door
Scratching the door	The cat moves repeatedly the paws against the door
Sniffing the door	The cat seems to smell by inhaling air under the door or on the door
Play	Object—The cat manipulates an object with its paws in an apparently playful mannerLocomotor—The cat plays with its own body, runs and jumps or rolls around
Play person/speaker	The cat directs elements of play behaviour at the observer or the source, attempting to interact. This comprises attempts to chase movements of the Reader, pouncing, and trying to catch the Reader or the speaker with the paw
Self-grooming	The cat cleans itself by licking, scratching, or biting the fur
Rolling	The cat rubs the back against the ground, with the belly exposed and all paws in the air. The cat may continue rolling repeatedly from side to side.
Knead	The cat presses and stretches its paws on a surface, alternating feet
Resting/sleeping	The cat is lying inactive, resting with the eyes closed or open but with the head touching the ground
Feeding/drinking	The cat ingests food (or other edible substances) by means of chewing with the teeth and swallowing. The cat ingests water by lapping up with the tongue and swallowing
Hide/Attempt to hide	The cat is withdrawing/fleeing from the field of vision of the Reader showing signs of fear
Hissing *	A drawn-out, low intensity hissing sound produced by rapid expulsion of air from the cat
Meowing *	Persistent and distinct sound, not related to anticipation of food
Yawning *	The cat opens its mouth widely while inhaling, then closes mouth while exhaling deeply
Other behaviours	The cat performs other behaviours than those listed above (e.g., cat sniffs the wall)
Not visible	The behaviour of the cat is not visible from the outside of the enclosure

* Recorded as events due to their short duration.

**Table 3:** Number and percentage of dogs (*N* = 14) and cats (*N* = 21) showing a specific behaviour during the two testing conditions (P+: person present or P−: person not present during the play back of the audio book).

	Number and Percentage of Dogs				Number and Percentage of Cats		
Dog Behaviour	P+ *N* (%)	P− *N* (%)	Total *N* (%)	Cat Behaviour	P+ *N* (%)	P− *N* (%)	Total *N* (%)
Stay in Section 1	7 (50)	9 (64)	10 (71)	Standing	15 (71)	16 (76)	19 (91)
Stay in Section 2 floor	11 (79)	10 (71)	12 (86)	Sitting	18 (86)	20 (95)	21 (100)
Stay in Section 2 bed	10 (71)	8 (57)	11 (79)	Lying head up	15 (71)	16 (76)	18 (86)
Stay in Section 3	8 (57)	9 (64)	10 (71)	Lying head down	12 (57)	6 (29)	14 (67)
Lying resting	4 (29)	5 (36)	7 (50)	Active/in locomotion	15 (71)	17 (81)	21 (100)
Lying attentive	13 (93)	13 (93)	14 (100)	Location front	19 (91)	17 (81)	20 (95)
Walking	12 (86)	11 (79)	13 (93)	Location back	15 (71)	16 (76)	19 (91)
Standing	12 (86)	10 (71)	13 (93)	Location up	4 (19)	10 (48)	10 (48)
Sitting	10 (71)	10 (71)	12 (86)	Interact source	3 (14)	5 (24)	7 (33)
Looking at auditory source	14 (100)	12 (86)	14 (100)	Interact person	6 (29)	0 (100)	6 (29)
Ears back	12 (86)	12 (86)	13 (93)	Rubbing the door	5 (24)	0 (0)	5 (24)
Exploring	7 (50)	6 (43)	10 (71)	Scratching the door	5 (24)	0 (0)	5 (24)
Self-grooming	6 (43)	6 (43)	9 (64)	Play	7 (33)	4 (19)	10 (48)
Panting	3 (21)	3 (21)	4 (29)	Play person	1 (5)	0 (100)	1 (5)
Lip lick	8 (57)	7 (50)	9 (64)	Self-grooming	10 (48)	12 (57)	16 (76)
Yawn	4 (29)	3 (21)	6 (43)	Feeding/drinking	6 (27)	3 (14)	7 (33)
Bark	6 (43)	5 (36)	8 (57)	Resting/sleeping	9 (43)	6 (29)	13 (62)
				Other behaviours	20 (95)	21 (100)	21 (100)
				Meowing	8 (38)	7 (33)	9 (43)
				Yawning	7 (33)	2 (10)	7 (33)
**Rare behaviours excluded from further analyses:**				**Rare behaviours excluded from further analyses:**			
Stereotypies	1 (7)	0 (0)	1 (7)	Rolling	0 (0)	0 (0)	0 (0)
Avoiding	0 (0)	0 (0)	0 (0)	Knead	0 (0)	0 (0)	0 (0)
Cowering	0 (0)	0 (0)	0 (0)	Using the litterbox	0 (0)	0 (0)	0 (0)
Tail lowered	0 (0)	0 (0)	0 (0)	Hide/Attempt to hide	0 (0)	0 (0)	0 (0)
Manipulating the environment	3 (21)	2 (14)	4 (29)	Hissing	0 (0)	0 (0)	0 (0)
Show back	1 (7)	1 (7)	2 (14)				
Shaking	2 (14)	0 (0)	2 (14)				
Stretching	0 (0)	0 (0)	0 (0)				

**Table 4:** Overview of the dog behaviour results, with mean, standard deviation (S.D.), minimum (Min), lower quartile (25%), median (Med), upper quartile (75%) and maximum (Max). Wilcoxon tests were used to compare the behaviours of the dogs between the two testing conditions (P+: person present or P−: person not present during the play back of the audio book). Significant differences are depicted in bold.

	Condition	Mean	S.D.	Min	25%	Med	75%	Max	Z	*p*
% Section 1	P+	15	28	0	0	1	25	100	−0.255	0.799
	P−	10	16	0	0	3	12	50		
% Section 2 floor	P+	20	30	0	0	6	27	100	−1.490	0.136
	P−	40	37	0	0	46	75	95		
**% Section 2 bed**	**P+**	**53**	**44**	**0**	**0**	**65**	**94**	**100**	**−1.988**	**0.047**
	**P−**	**24**	**42**	**0**	**0**	**2**	**17**	**100**		
% Section 3	P+	13	20	0	0	2	26	66	−1.784	0.074
	P−	25	34	0	0	7	38	100		
% Lying resting	P+	16	33	0	0	0	3	99	−0.676	0.499
	P−	8	18	0	0	0	4	61		
% Lying attentive	P+	43	37	0	11	31	89	98	−1.601	0.109
	P−	66	30	0	48	71	89	100		
% Walking	P+	5	8	0	1	2	5	30	−1.328	0.184
	P−	3	3	0	0	2	4	10		
% Standing	P+	16	18	0	5	8	32	59	−1.013	0.311
	P−	11	17	0	0	6	12	65		
% Sitting	P+	15	23	0	0	2	28	64	−0.078	0.937
	P−	11	15	0	0	3	17	42		
**% Looking at source**	**P+**	**8**	**6**	**1**	**2**	**7**	**12**	**19**	**−2.919**	**0.004**
	**P−**	**2**	**2**	**0**	**0**	**2**	**4**	**7**		
% Ears back	P+	18	25	0	1	8	22	88	−1.083	0.279
	P−	17	30	0	0	5	16	91		
% Exploring	P+	2	5	0	0	0	2	20	−0.255	0.799
	P−	1	3	0	0	0	1	9		
% Self-grooming	P+	0	1	0	0	0	1	2	−0.533	0.594
	P−	2	4	0	0	0	1	12		
% Panting	P+	6	16	0	0	0	0	58	−0.730	0.465
	P−	3	7	0	0	0	0	20		
Lip lick/min	P+	0.7	1.0	0.0	0.0	0.3	1.1	2.5	−0.889	0.374
	P−	0.5	0.8	0.0	0.0	0.1	0.8	2.6		
Yawn/min	P+	0.1	0.2	0.0	0.0	0.0	0.1	0.8	−0.105	0.917
	P−	0.1	0.3	0.0	0.0	0.0	0.0	0.9		
Bark/min	P+	1.1	2.9	0.0	0.0	0.0	0.1	10.6	0.000	1.000
	P−	1.0	2.9	0.0	0.0	0.0	0.2	10.7		

**Table 5:** Spearman rank correlations between “% Section 2 bed” and “% Looking at auditory source” and all other behaviours recorded in dogs during the P+ condition only (*N* = 14). Significant correlations are depicted in bold, tendencies in italics.

		% Section 2 Bed	% Looking at Auditory Source
% Section 1	r_s_	*−0.48 ^t^*	0.22
**% Section 2 floor**	**r_s_**	−0.62 *	0.36
% Section 2 bed	r_s_	---	−0.27
% Section 3	r_s_	*−0.42 ^t^*	0.38
% Lying resting	r_s_	0.22	−0.30
% Lying attentive	r_s_	0.24	0.14
% Walking	r_s_	*−0.48 ^t^*	0.40
**% Standing**	**r_s_**	−0.57 *	0.11
% Sitting	r_s_	−0.40	0.35
% Looking at auditory source	r_s_	−0.27	---
% Ears back	r_s_	−0.16	0.23
% Exploring	r_s_	−0.24	0.17
% Self-grooming	r_s_	−0.38	0.30
**% Panting**	**r_s_**	−0.05	0.72 **
**Lip lick/min**	**r_s_**	0.10	0.58 *
Yawn/min	r_s_	−0.04	*0.47 ^t^*
Bark/min	r_s_	0.31	0.06

** *p* < 0.01, * *p* > 0.01 ≤ 0.05, *^t^ p* > 0.05 ≤ 0.1.

**Table 6:** Overview of the cat behaviours, with mean, standard deviation (S.D.), minimum (Min), lower quartile (25%), median (Med), upper quartile (75%) and maximum (Max). Wilcoxon tests were used to compare the behaviours of the cats between the two testing conditions (P+: person present, P−: person not present during the play back of the audio book). Significant differences are depicted in bold, tendencies in italics.

	Condition	Mean	S.D.	Min	25%	Med	75%	Max	Z	*p*
% Standing	P+	11.79	18.42	0.00	0.00	1.77	22.76	69.57	−1.449	0.147
	P−	7.76	11.02	0.00	0.13	1.53	12.56	33.99		
% Sitting	P+	40.21	30.82	0.00	20.22	35.62	58.78	100.0	−0.504	0.614
	P−	43.51	27.26	0.00	22.86	45.91	63.53	98.53		
% Lying head up	P+	26.79	26.41	0.00	0.00	17.19	36.75	98.03	−0.762	0.446
	P−	34.23	32.01	0.00	3.12	27.70	61.97	100.0		
% Lying head down	P+	12.65	24.29	0.00	0.00	2.06	7.56	75.27	−1.287	0.198
	P−	7.97	22.47	0.00	0.00	0.00	2.83	89.64		
% Active locomotion	P+	8.56	11.91	0.00	0.00	2.87	13.93	31.43	−0.782	0.434
	P−	6.53	7.28	0.00	1.13	3.53	8.09	22.61		
% Loc front	P+	54.32	34.21	0.00	29.72	53.27	99.23	100.0	−0.966	0.334
	P−	46.47	37.24	0.00	8.04	44.42	77.91	100.0		
% Loc back	P+	39.38	33.06	0.00	0.00	42.01	69.61	100.0	−0.201	0.841
	P−	38.20	35.36	0.00	3.60	41.42	61.92	100.0		
% Loc up	P+	6.30	22.02	0.00	0.00	0.00	0.00	100.0	*−1.955*	*0.051*
	P−	15.33	30.35	0.00	0.00	0.00	10.52	100.0		
% Interact source	P+	0.13	0.39	0.00	0.00	0.00	0.00	1.70	−1.521	0.128
	P−	0.57	1.75	0.00	0.00	0.00	0.00	7.97		
**% Rubbing door**	**P+**	**0.13**	**0.30**	**0.00**	**0.00**	**0.00**	**0.00**	**0.94**	**−2.023**	**0.043**
	**P−**	**0.00**	**0.00**	**0.00**	**0.00**	**0.00**	**0.00**	**0.00**		
**% Scratching door**	**P+**	**0.10**	**0.31**	**0.00**	**0.00**	**0.00**	**0.00**	**1.40**	**−2.023**	**0.043**
	**P−**	**0.00**	**0.00**	**0.00**	**0.00**	**0.00**	**0.00**	**0.00**		
% Play	P+	3.76	10.41	0.00	0.00	0.00	1.72	44.30	−0.153	0.878
	P−	4.24	12.93	0.00	0.00	0.00	0.00	56.31		
% Self-grooming	P+	2.65	5.03	0.00	0.00	0.00	2.90	18.50	−0.879	0.379
	P−	10.85	24.94	0.00	0.00	0.29	3.07	89.27		
% Feeding/Drinking	P+	8.52	17.52	0.00	0.00	0.00	12.05	65.60	−1.521	0.128
	P−	1.42	5.41	0.00	0.00	0.00	0.00	24.64		
% Resting/Sleeping	P+	9.82	24.41	0.00	0.00	0.00	3.59	100.00	−0.245	0.807
	P−	7.28	20.32	0.00	0.00	0.00	3.90	89.57		
% Other	P+	70.95	27.64	0.00	54.15	80.26	93.56	100.0	−0.33	0.741
	P−	73.84	31.32	7.36	62.01	89.72	95.70	98.80		
Meow/min	P+	0.50	1.19	0.00	0.00	0.00	0.30	4.40	*−1.779*	*0.075*
	P−	0.23	0.70	0.00	0.00	0.00	0.20	3.23		
**Yawn/min**	**P+**	**0.04**	**0.07**	**0.00**	**0.00**	**0.00**	**0.10**	**0.20**	**−2.371**	**0.018**
	**P−**	**0.01**	**0.03**	**0.00**	**0.00**	**0.00**	**0.00**	**0.10**		

**Table 7:** Spearman rank correlations between “% Watch source”, “% Rubbing door”, “% Scratching door” and “% Yawning” and the other behaviours recorded in cats during the P+ condition. Significant correlations are depicted in bold, tendencies in italics.

		% Rubbing Door	% Scratching Door	Yawning/Minute
% Standing	r_s_	0.10	0.26	*−0.39 ^t^*
% Sitting	r_s_	−0.05	−0.07	0.30
% Lying head up	r_s_	0.23	0.35	−0.07
% Lying head down	r_s_	0.02	−0.03	0.03
% Active locomotion	r_s_	0.12	0.35	−0.29
**% Loc front**	**r_s_**	0.13	−0.13	0.61 **
**% Loc back**	**r_s_**	−0.02	0.28	−0.48 *
% Loc up	r_s_	−0.27	−0.27	−0.33
**% Interact source**	**r_s_**	0.01	0.40	0.62 **
**%Interact person**	**r_s_**	0.37	0.57 **	0.40
% Rubbing door	r_s_	---	0.25	0.22
% scratching door	r_s_	0.25	---	0.30
% Play	r_s_	−0.00	0.43	0.25
**%Play person**	**r_s_**	0.30	0.49 *	0.35
% Self-grooming	r_s_	0.05	0.29	0.13
%Feeding/drinking	r_s_	−0.16	0.11	−0.00
% Resting/Sleeping	r_s_	−0.18	−0.07	−0.02
% Other	r_s_	0.18	−0.26	−0.22
**Meow/min**	**r_s_**	0.57 **	−0.07	0.17
Yawning/min	r_s_	0.22	0.30	---

** *p* < 0.01, * *p* > 0.01 ≤ 0.05, *^t^ p* > 0.05 ≤ 0.1.

## Data Availability

The data presented in this study are available in Supplementary Materials Excel S1: Study Data (data_tuozzi_arhant.et.al.xlsx).

## Supplementary Materials

The following are available online at https://www.mdpi.com/2076-2615/11/2/406/s1, Excel S1: Study Data (data_tuozzi_arhant.et.al.xlsx).
